# Comprehensive Bioinformatic Analysis of Epithelial-Mesenchymal Transition (EMT) Network-Related microRNAs As Candidate Signatures in Prostate Adenocarcinoma

**DOI:** 10.7759/cureus.86467

**Published:** 2025-06-20

**Authors:** Gautam Prasad, Dilutpal Sharma, Anveshika Manoj, Mohammad Kaleem Ahmad

**Affiliations:** 1 Biochemistry, King George's Medical University, Lucknow, IND

**Keywords:** emt, gene, in-silico, mirna, prostate cancer

## Abstract

Prostate cancer is an adenocarcinoma that involves epithelial-mesenchymal transition (EMT) for metastasis. To uncover novel insights into the development of prostate tumors and to identify important genes and putative microRNAs (miRs) for patient care, this study performed an in-depth bioinformatics analysis using dbDEMC3.0 (Zhejiang University, Hangzhou, China), MIENTURNET (University of Rome Tor Vergata, Rome, Italy), and DIANA-miTED (University of Thessaly, Thessaly, Greece) to explore miRs regulating tumorigenesis, proliferation, and potential therapeutic targets. A total of 373 differently expressed miRs were examined in this study, of which 87 had significant upregulation and 85 had significant downregulation. Our results from the MIENTURNET software showed that miR-141-3p, miR-200a-3p, miR-200b-3p, miR-200c-3p, miR-203a-3p, miR-429, miR-34a-5p, and miR-509-3-5p interact with the transcription factors CDH1, CDH2, SNAI1, ZEB1, and ZEB2, which play a significant role in the core EMT regulatory network. The Encyclopedia of RNA Interactomes (ENCORI) miR-target interaction co-expression analysis observed that miR-34a-5p had a strong interaction with CDH1 as compared to other genes. The results of DIANA-plasmiR analysis showed that miR-34a-5p is a useful prognostic and diagnostic biomarker. Our results suggest that this study advances our knowledge of the molecular mechanism underlying prostate adenocarcinoma and that the interaction between the EMT gene and differentially expressed miR (DEmiR) in prostate adenocarcinoma may represent a target for prostate cancer diagnosis and treatment.

## Introduction

The Global Cancer Observatory reports that 1.5 million new cases of prostate cancer were reported in 2022, thereby becoming the second most common cancer worldwide and the fifth highest cause of cancer-related deaths among men [1]. This rise in incidence is due to the growing adoption of the prostate-specific antigen (PSA) test for prostate cancer diagnosis. Unfortunately, this test's lack of specificity results in a high percentage of false positives and the diagnosis of indolent diseases [2]. The steady death rate suggests that this has raised the burden of prostate cancer illness and caused overtreatment of patients without increasing overall survival. Furthermore, within five years of receiving radical prostatectomy (RP), one of the first-line curative therapies for localized prostate cancer, 15-45% of patients develop biochemical recurrence (BCR). BCR is considered an early occurrence that signals the progression of the disease, even though it doesn't always mean a clinical recurrence [3].

These problems highlight how important it is to reliably and accurately differentiate between indolent and aggressive diseases in order to avoid over-treating patients and provide prostate cancer management with feasible treatment strategies. Potential substitute molecular markers for prostate cancer have been studied, including microRNA (miR). miR, which are small non-coding RNAs, possess the capacity to adversely regulate the expression of genes that are post-transcribed. Their mode of action involves binding to complementary sequences in the 3′ untranslated region (3′ UTR) of target mRNAs through a section known as the “seed sequence,” thereby preventing the target mRNAs from being translated [4]. Many physiological, cellular, and developmental processes are regulated by these molecules, and their dysregulation has been connected to a number of diseases [5]. Prostate cancer is among the many cancers that have been shown to exhibit differentiating miR expression levels between normal tissues and tumors [6]. Additional benefits of miRs as biomarkers are their great stability in storage, their consistent and profuse expression in vivo, and their detection in biofluids such as blood, urine, and saliva [7].

The miR-34 family controls epithelial-mesenchymal transition (EMT) through the tumor suppressor p53 and EMT-associated transcription factors (EMT-TFs) [8]. Cancer cells have the ability to become migratory and invasive during the growth of a tumor by initiating an EMT phase, which may result in distant metastases. EMT transcription factors such as snail, twist, and slug lead to the downregulation of E-cadherin expression, which increases tumor invasiveness and triggers the EMT [9].

Loss of expression of CDH1, a calcium-dependent transmembrane protein crucial in maintaining cell integrity and polarity, leads to decreased cell adhesion and increased invasiveness [10].

In this study, we aimed to identify EMT-related miRNA signatures in prostate adenocarcinoma through comprehensive bioinformatic analysis, validate their interactions with core EMT genes (e.g., CDH1, CDH2, SNAI1, ZEB1, ZEB2), and evaluate their potential as diagnostic or prognostic biomarkers. Our findings propose a novel regulatory loop involving miR-34a-5p/CDH1 in prostate adenocarcinoma, which may serve as a promising biomarker or a potential therapeutic target in future research.

## Materials and methods

Identification of differentially expressed miR (DEmiR)

Following the manufacturer’s guidelines, miRNA expression profiling was performed using Agilent miRNA Microarray V2 arrays (EXP00101; Agilent Technologies, Santa Clara, CA, USA) on samples from 99 prostate cancer patients and 28 control subjects [11]. Raw microarray data were processed through quantile normalization using the robust multi-array average (RMA) algorithm, followed by log_2_ transformation. For quality control, probes with detection p-values ≥ 0.05 in > 80% of samples were filtered out, and miRNAs detected in fewer than 20% of samples were excluded as low-expression signals. Differential expression analysis was conducted using the Limma package in R (The R Foundation for Statistical Computing, Vienna, Austria), with statistically significant miRNAs identified under two thresholds: a primary cutoff of absolute log_2_ fold change (|log_2_FC|) > 0.5 and a stringent validation cutoff of |log_2_FC| > 1.0, both with an adjusted p-value (Benjamini-Hochberg false discovery rate (FDR)) < 0.05. This analysis yielded 373 DEmiRs for further investigation.

Prediction of targets for DEmiR

The target genes of the miR that were differently expressed were found using the MIENTURNET program (University of Rome Tor Vergata, Rome, Italy). The potential of these widely recognized databases is to precisely predict miRNA target genes.

Functional enrichment analyses

The web-based tool MIENTURNET (http://userver.bio.uniroma1.it/apps/mienturnet/) was employed to identify miRNA-target gene interactions and predict their associated molecular functions, biological processes, cellular components, and pathways. For enrichment analyses performed using FunRich software (Dr. Suresh Mathivanan, La Trobe University, Australia), we applied the standard statistical cutoff of p < 0.05, consistent with conventional thresholds used in similar studies.

Predicted miRNAs-mRNAs network construction

The MultiMiR software (University of Colorado, Aurora, Colorado, USA) was used for predicting the target genes of DEmiR. MIENTURNET, the regulatory network of predicted miR-mRNAs, has been constructed, and the strongest 10 mRNAs and miRs with the highest degree were discovered. MIENTURNET was run with default parameters (TargetScan, miRDB, and miRTarBase as source databases; interaction significance: p < 0.05).

Analysis of precise miRNA expression

The interacting miR-141-3p, miR-200a-3p, miR-200b-3p, miR-200c-3p, miR-203a-3p, miR-429, miR34a-5p, and miR-509-3-5p from MIENTURNET that are involved in the core EMT regulation network have been identified. The DIANA program is an internet-based web server that facilitates the analysis of miR/mRNA expression data obtained from next-generation sequencing experiments. It has advanced overrepresentation analysis capabilities and helps identify significant miRs within the data. The analysis of miR expression in prostatic cancer cell lines (PC-3, DU145) and normal cell line (SH-SY5Y) was conducted in human prostate glands with DIANA-miTED (https://dianalab.e-ce.uth.gr/mited/#/; University of Thessaly, Thessaly, Greece). Furthermore, these miR expressions were confirmed in 495 prostate cancer patients and 52 normal samples with the use of the Encyclopedia of RNA Interactomes (ENCORI) database.

Targeted gene expression analysis

The targeted gene for EMT expression analysis was analysed using the Kaplan-Meier (KM) plotter software (Semmelweis University, Budapest, Hungary). By using the Gene Expression Profiling Interactive Analysis (GEPIA) server (http://gepia.cancer-pku.cn/) on 492 prostate cancer samples and 152 normal samples, we further validated the mRNA expression level of EMT genes: CDH1, CDH2, SNAI1, ZEB1, and ZEB2 in prostate cancer.

Biomarker assessment

The DIANA-plasmiR database (https://dianalab.e-ce.uth.gr/plasmir/#/) was utilized to analyze diagnostic and prognostic biomarker entries for miR-141-3p, miR-200a-3p, miR-200b-3p, miR-200c-3p, miR-203a-3p, miR-429, miR-34a-5p, and miR-509-3-5p. The results were subsequently validated using 10-fold cross-validation with 95% confidence intervals.

Validation of miR-target interaction co-expression analysis

ENCORI (https://rnasysu.com/encori/) was used for the validation of screened miR-target interaction co-expression analysis of miR-34a-5p versus EMT gene: (A) CDH1, (B) CDH2, (C) ZEB1, (D) ZEB2, and (E) SNAI1 in 495 prostate cancer patient samples. Key findings of the miR-34a-5p/CDH1 interaction were cross-validated using ENCORI (Pearson correlation, p < 0.01) and GEPIA based on The Cancer Genome Atlas-Prostate Adenocarcinoma (TCGA-PRAD) cohort.

Analysis concerning the survival of crucial genes

Transcriptional expression analysis was performed using PRAD samples (n = 211) and normal prostate tissues (n = 408) from the TCGA database (https://portal.gdc.cancer.gov/), analyzed through the GEPIA web server. For prognostic evaluation, the KM plotter tool (https://kmplot.com/analysis/) was employed to assess the predictive value of significant genes in PRAD patients. KM survival curves were generated using optimal expression cutoffs (determined by receiver operating characteristic (ROC) analysis) to stratify patients into high and low CDH1 expression groups. These cutoffs were calculated using maximally selected rank statistics (maxstat R package). Together, these analyses confirmed the clinical significance of key genes in prostate cancer progression.

For technical validation, all analyses incorporated cross-platform normalization (quantile normalization for microarray data and transcripts per million (TPM) for RNA-seq), along with multiple testing correction using the Benjamini-Hochberg FDR, and independent validation in both TCGA and GEO (GSE21036) cohorts.

## Results

Identification of DEmiRs

**Figure 1:**
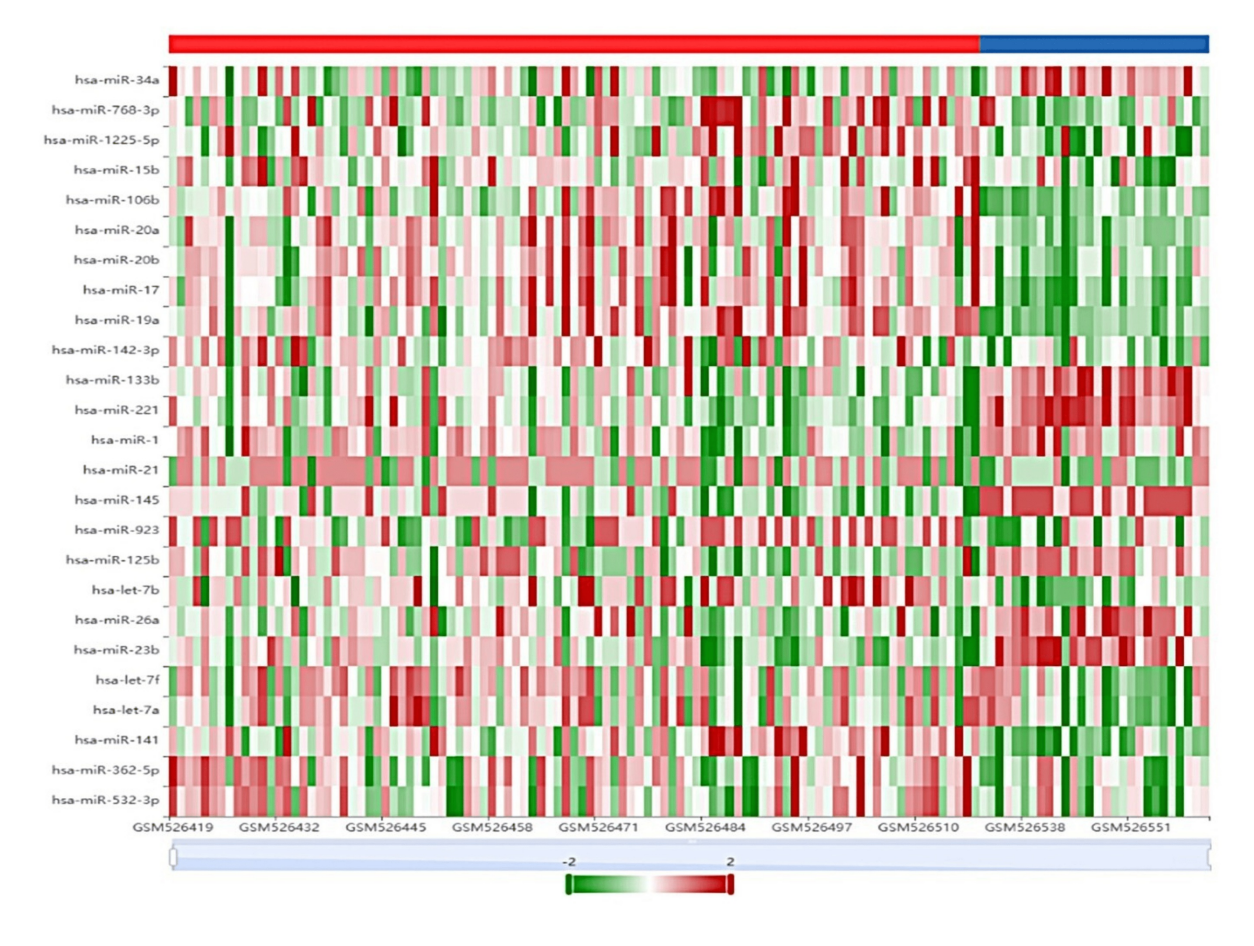
Heatmap of differentially expressed miRNA in prostate cancer patients and control sample by using dbDEMC software.

A total of 373 miRs were screened in prostate cancer patients, out of which 87 miRs were highly elevated and 85 significantly downregulated when compared to the control sample (Figure 1 and Table 1).

**Table 1:** Differential expression of miRNA in prostate cancer patients and control sample by using Agilent-019118 Human miRNA Microarray 2.0 G4470B quantified by Limma package in R software.

miRNA ID	Cancer Type	log Fold Change	Average Expression	T-value	P-value	Adjusted P-value	Status
hsa-miR-99b	Prostate cancer	-0.75	8.62	-6.09	9.64e-9	2.25e-7	Down
hsa-miR-27b	Prostate cancer	-0.58	12.19	-5.86	2.98e-8	6.54e-7	Down
hsa-miR-141	Prostate cancer	1.09	4.44	5.64	8.98e-8	1.86e-6	Up
hsa-miR-29a	Prostate cancer	-0.59	4.32	-5.62	9.61e-8	1.89e-6	Down
hsa-miR-143	Prostate cancer	-0.94	11.7	-5.59	1.12e-7	2.09e-6	Down
hsa-miR-602	Prostate cancer	0.77	3.99	5.52	1.57e-7	2.79e-6	Up
hsa-miR-133b	Prostate cancer	-1.06	9.67	-5.47	1.99e-7	3.37e-6	Down
hsa-miR-106b	Prostate cancer	0.53	10.33	5.43	2.37e-7	3.84e-6	Up
hsa-miR-17	Prostate cancer	0.61	10.11	5.26	5.11e-7	7.94e-6	Up
hsa-miR-200c	Prostate cancer	0.77	11.84	5.23	5.92e-7	8.15e-6	Up
hsa-miR-141	Prostate cancer	0.83	13.06	5.23	5.99e-7	8.15e-6	Up
hsa-miR-19a	Prostate cancer	0.67	9.75	5.22	6.06e-7	8.15e-6	Up
hsa-miR-886-3p	Prostate cancer	-1.01	8.41	-5.22	6.12e-7	8.15e-6	Down
hsa-miR-32	Prostate cancer	1.12	6	5.18	7.42e-7	9.54e-6	Up
hsa-miR-23b	Prostate cancer	-0.56	12.83	-5.16	7.97e-7	9.91e-6	Down

Analysis of functional and pathway enrichment for designated target genes

**Figure 2:**
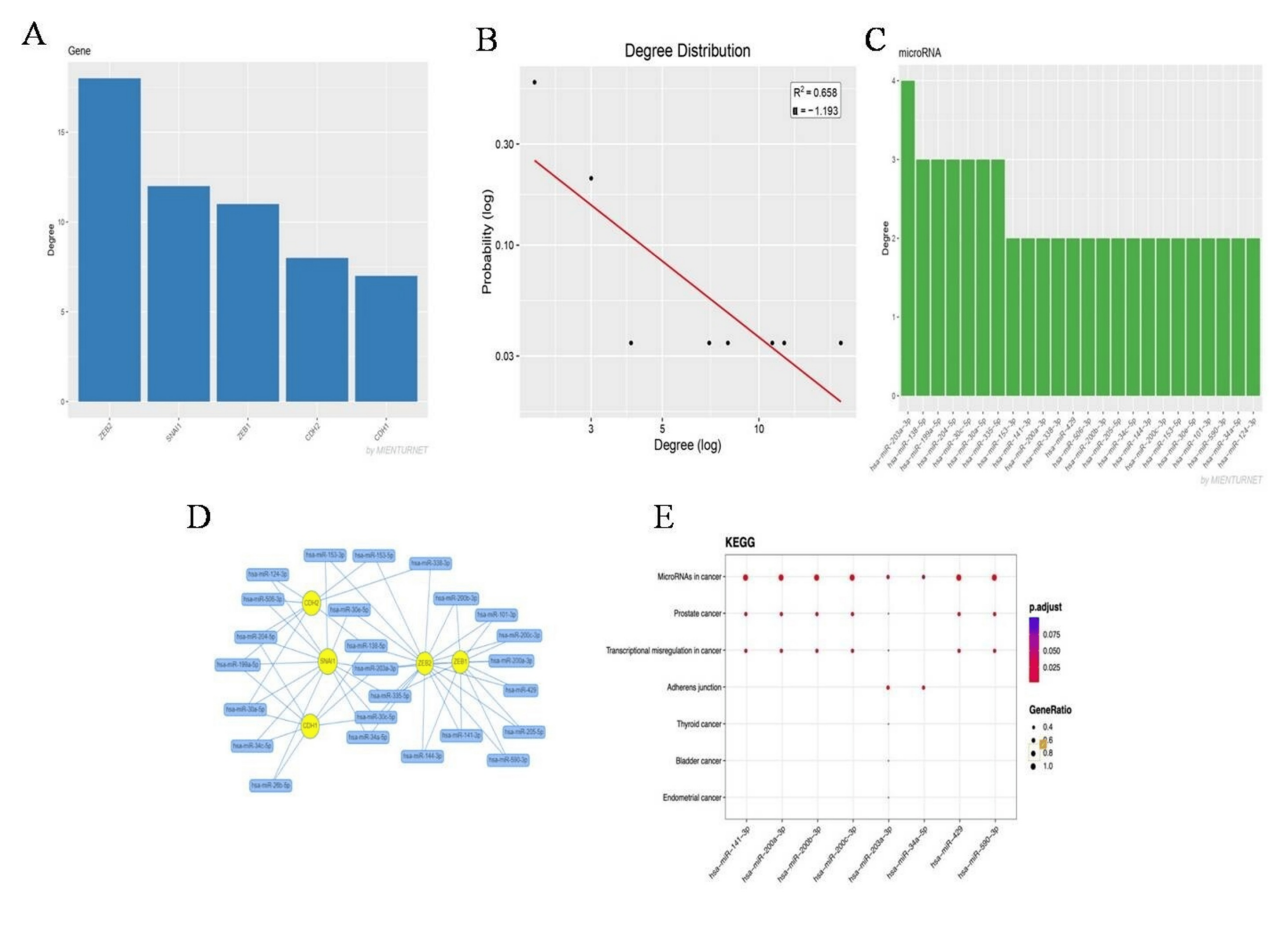
Output of the MIENTURNET web tool based on gene input. (A) Network degree plot of target genes (top-left). (B) Node degree distribution on a double logarithmic scale (log-log plot), where the straight line in the top-middle represents the power-law fit. (C) Network degree plot for miRNAs (top-right). (D) Visual representation of the miRNA-target interaction network, where target genes are shown as yellow circles and miRNAs as blue boxes. (E) Functional enrichment analysis dot plot for target genes of selected miRNAs. The X-axis shows the selected miRNAs, while the Y-axis displays annotation categories (e.g., KEGG pathways). Dot size indicates the gene ratio (the proportion of miRNA target genes enriched in each category), and dot color represents adjusted p-values calculated using the FDR method. KEGG: Kyoto Encyclopedia of Genes and Genomes; FDR: false discovery rate

MEINTURNET data demonstrates that the various miR-141-3p, miR-200a-3p, miR-200b-3p, miR-200c-3p, miR-203a-3p, miR-429, miR-34a-5p, and miR-509-3-5p interact with the gene that specifies in the EMT (CDH1, CDH2, SNAI1, ZEB1, ZEB2) (Figure 2D). Network analysis of the gene degree plot (Figure 2A) and the miR degree plot (Figure 2C) showed that, when compared to other genes, ZEB2 was targeted mostly by all miR, followed by SNAI1, ZEB1, CDH2, and CDH1. It has been demonstrated that, in comparison to other miRs, miR-203-3p plays a large role in the targeting of EMT genes. Functional enrichment analysis reveals that distinct miRs are involved in several cancer types, including prostate cancer (Figure 2E).

Expression analysis of targeted miR

**Figure 3:**
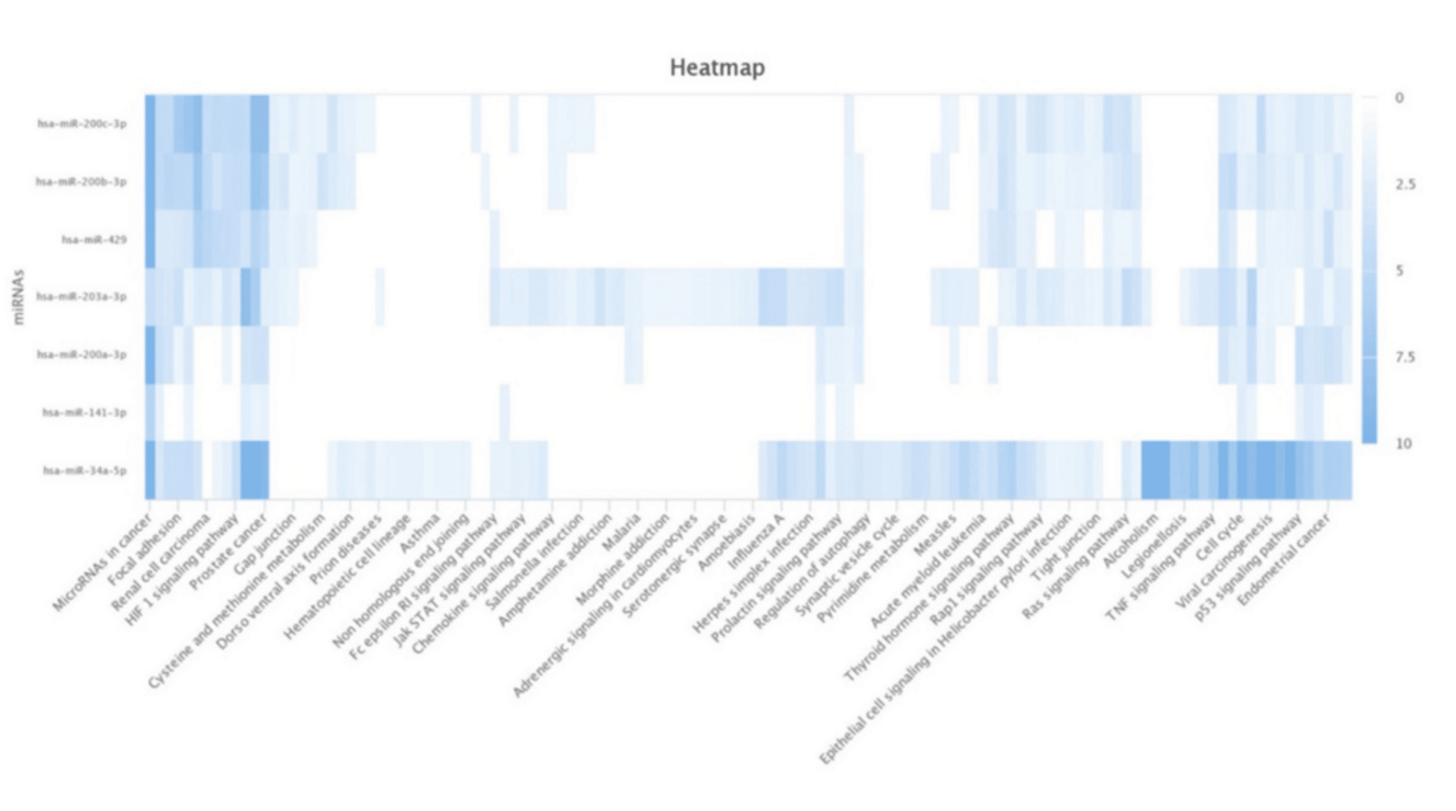
Heatmap showing the expression levels of miRNAs (miR-34a-5p, miR-141-3p, miR-200a-3p, miR-200b-3p, miR-200c-3p, miR-203a-3p, miR-429) across various cancer types and signaling pathways, including prostate cancer by using DIANA-miExTra v2.0.

**Figure 4:**
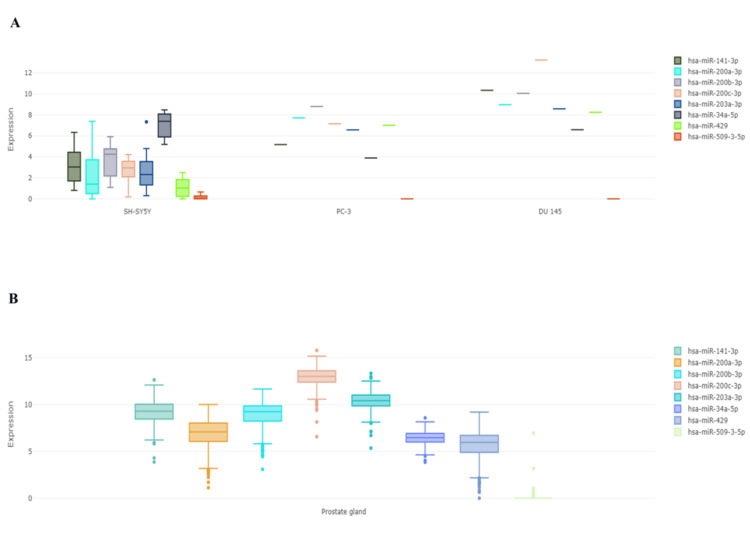
(A) Differential microRNA expression in the human prostate gland. (B) Differential microRNA expression in the normal cell line (SH-SY5Y) compared to prostate cancer cell lines (PC-3 and DU145) using DIANA-miTED database.

**Figure 5:**
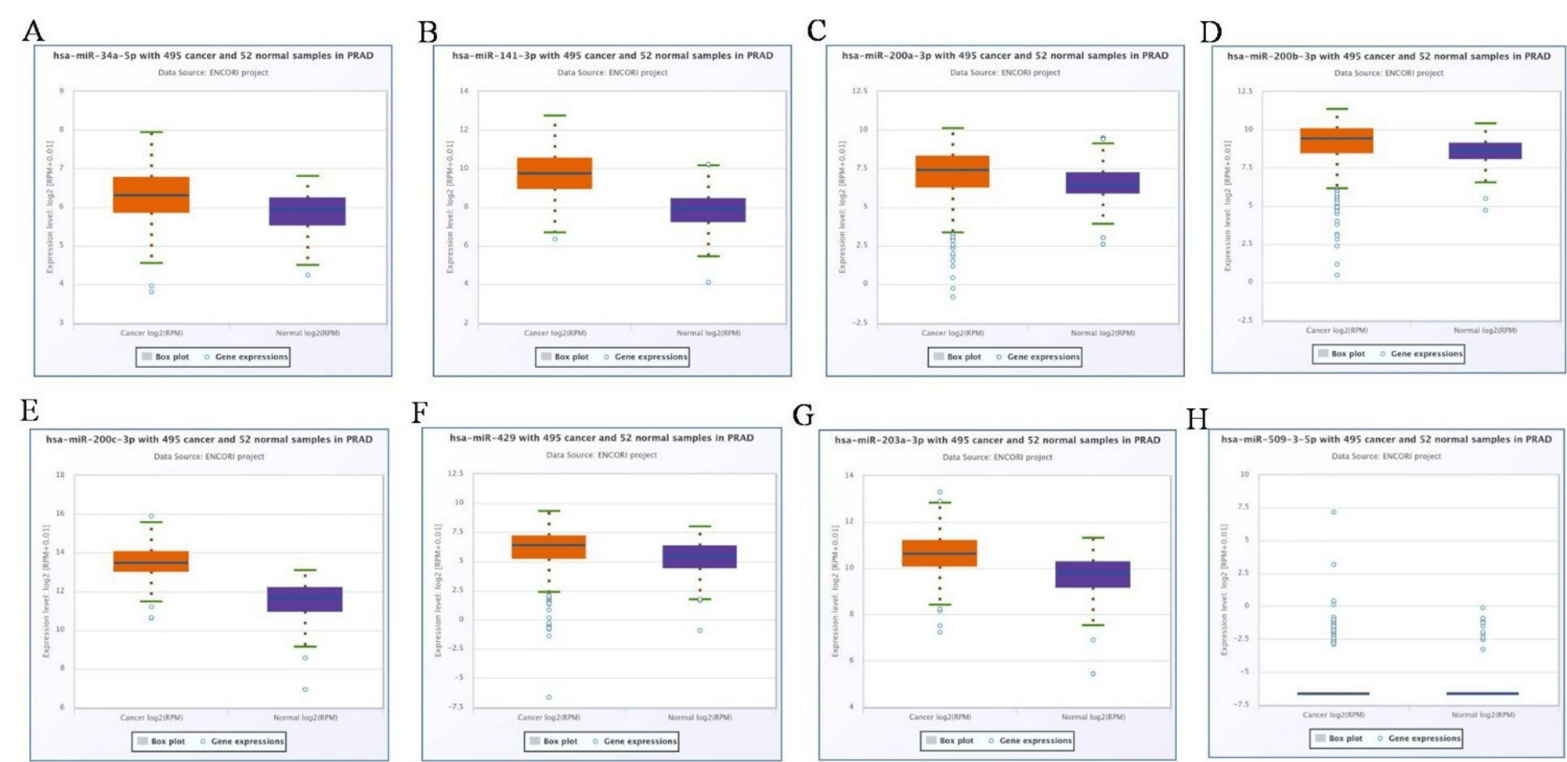
Validation of miRNA expression levels in (A) miR-34a-5p, (B) miR-141-3p, (C) miR-200a-3p, (D) miR-200b-3p, (E) miR-200c-3p, (F) miR-203a-3p, (G) miR-429, and (H) miR-509-3-5p. Box plots represent expression data from 495 prostate cancer patients (orange) and 52 normal samples (blue). A p-value < 0.05 was considered statistically significant.

It has been demonstrated that certain cancers and signalling pathways, including prostate cancer, express the EMT networks with miR-141-3p, miR-200a-3p, miR-200b-3p, miR-200c-3p, miR-203a-3p, miR-429, miR-34a-5p, and miR-509-3-5p (Figure 3). Furthermore, we examined the expression of miR in human prostate gland and PC-3, DU145, and SH-SY5Y cell lines. Our findings showed that miR-200c-3p and miR-141-3p were highly elevated, while miR-34a-5p was dramatically downregulated in the prostate gland (Figure 4A) and prostate cancer cell line (PC-3, DU145) compared to the normal cell line (SH-SY5Y) (Figure 4B). When comparing prostate cancer patients to a healthy control group, the data, which were subsequently verified by ENCORI, reveal that miR-34a-5p is downregulated, whereas miR-200c-3p and miR-141-3p are increased (Figure 5).

Biomarker prediction

**Figure 6:**
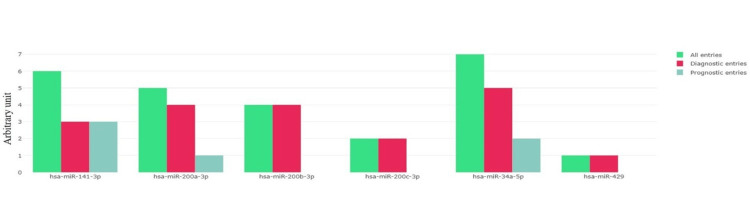
The figure depicts the number of total diagnostic and prognostic biomarker entries for selected microRNAs (miR-141-3p, miR-200a-3p, miR-200b-3p, miR-34a-5p, and miR-429), by using the DIANA-plasmiR database.

Using DIANA-plasmiR data on the basis of all the entries of miR-34a-5p,200a-3p, miR-200b-3p, miR-141-3p, miR-200c-3p, and miR-429, we found that miR-34a-5p might be a useful diagnostic and prognostic biomarker compared to others, followed by miR-200a-3p, miR-200b-3p, miR-141-3p, miR-200c-3p, and miR-429 (Figure 6).

Gene expression analysis

**Figure 7:**
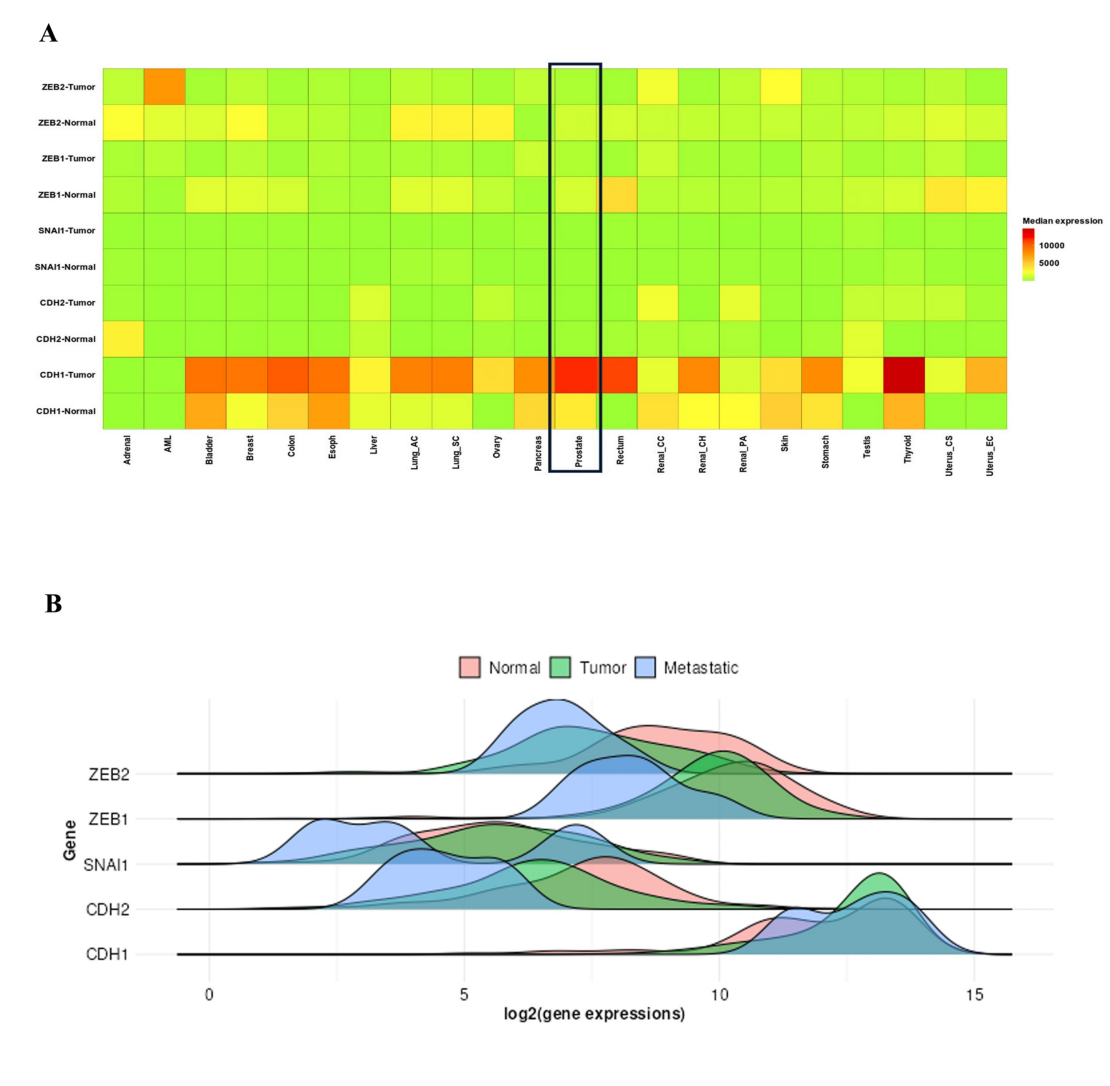
(A) Heatmap showing the expression of EMT-related genes (CDH1, CDH2, SNAI1, ZEB1, and ZEB2) across various cancer types, including prostate cancer. (B) Gene expression levels of CDH1, CDH2, SNAI1, ZEB1, and ZEB2 across normal, tumor, and metastatic stages.

**Figure 8:**
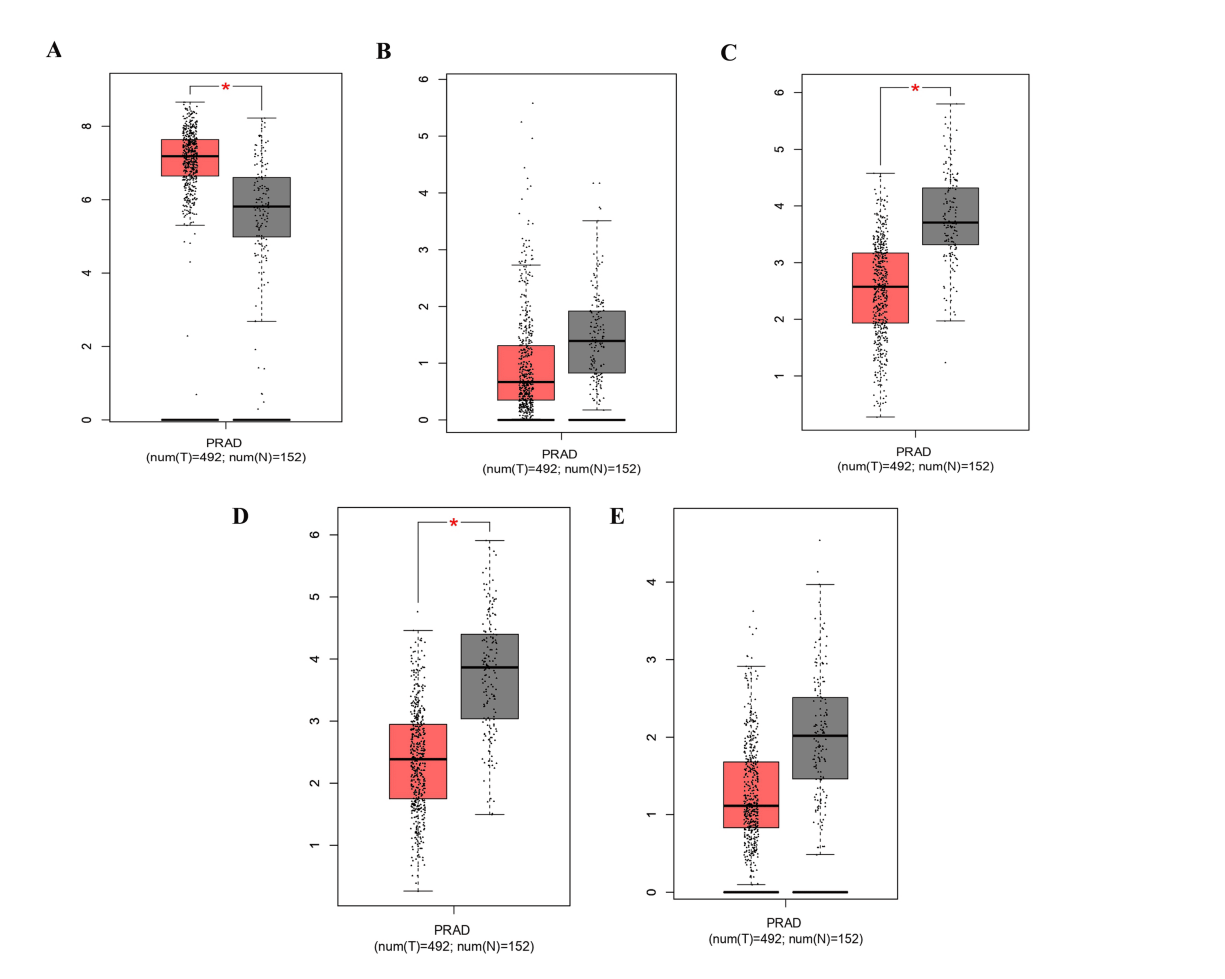
Validation of mRNA expression levels of (A) CDH1, (B) CDH2, (C) ZEB1, (D) ZEB2, and (E) SNAI1 in prostate cancer using the Gene Expression Profiling Interactive Analysis (GEPIA). Box plots are based on data from 492 prostate cancer samples (red) and 152 normal samples (grey). A p-value < 0.005 was considered statistically significant.

The EMT gene linked to prostate cancer was shown to have a specific expression level using a KM plotter. A heatmap analysis reveals that the expression of CDH1 is upregulated in breast, bladder, colon, lung, renal, and thyroid cancer, including prostate cancer, while in adrenal cancer and acute myeloid leukemia (AML), there is no change in the expression of the CDH1 gene. The other genes, CDH2, SNAI1, and ZEB1, showed no significant difference between tumor and healthy control, while the expression of ZEB2 is downregulated in breast, lung, and ovarian cancer patients compared to healthy controls, with no significant change observed in prostate cancer. To further elaborate on our result, in prostate cancer, a density plot was used, which confirms that there is no significant change in the expression level of CDH2, SNAI1, ZEB1, and ZEB2 genes in the tumor as compared to healthy controls. While the expression of CDH1 was dramatically elevated in the tumor as compared to normal (Figures 7A, 7B), the GEPIA software was utilized to further validate this, using data from 492 prostate cancer patients and 152 healthy controls. The results showed that the expression levels of CDH2, SNAI1, ZEB1, and ZEB2 were significantly downregulated in prostate cancer compared to controls, while the expression of CDH1 was significantly upregulated (Figure 8).

Co-expression of miRNA-target interactions and biomarker analysis prediction

**Figure 9:**
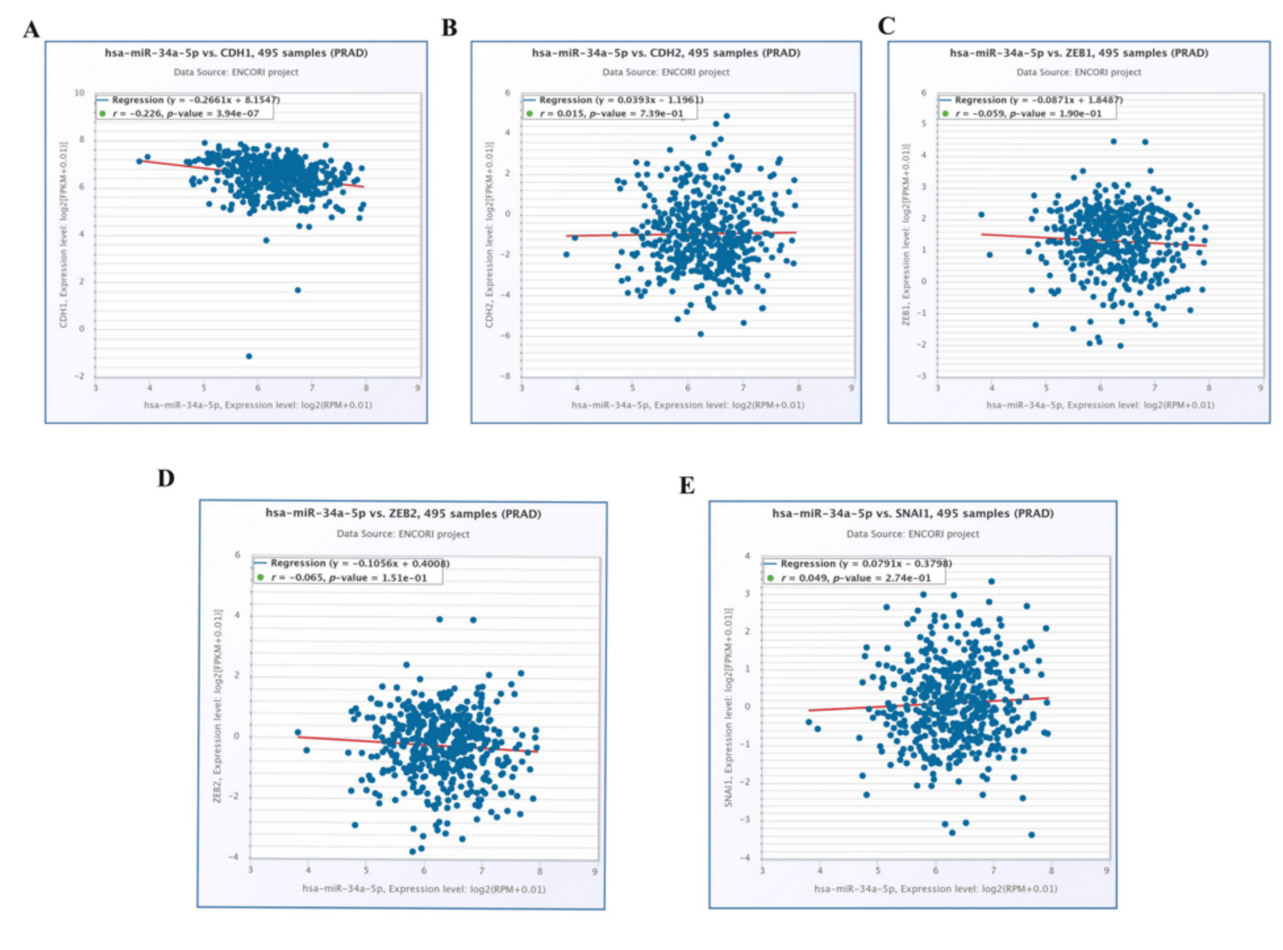
Validation of miRNA-target interaction co-expression analysis between miR-34a-5p and EMT-related genes: (A) CDH1, (B) CDH2, (C) ZEB1, (D) ZEB2, and (E) SNAI1 in 495 prostate cancer samples using the Encyclopedia of RNA Interactomes (ENCORI). The Y-axis represents gene expression levels as log_2_(FPKM + 0.01), and the X-axis represents microRNA expression levels as log_2_(RPM + 0.01). EMT: epithelial-mesenchymal transition

We found that miR-34a-5p is a useful diagnostic and prognostic biomarker by DIANA-plasmiR, so we further evaluated its connection with EMT genes (CDH1, CDH2, SNAI1, ZEB1, and ZEB2). The result demonstrated that a strong relationship exists between miR-34a-5p and CDH1 compared to other genes (CDH2, SNAI1, ZEB1, and ZEB2) (Figure 9).

Survival analysis and prognostic potential prediction of miR-34a-5p and CDH1 gene

**Figure 10:**
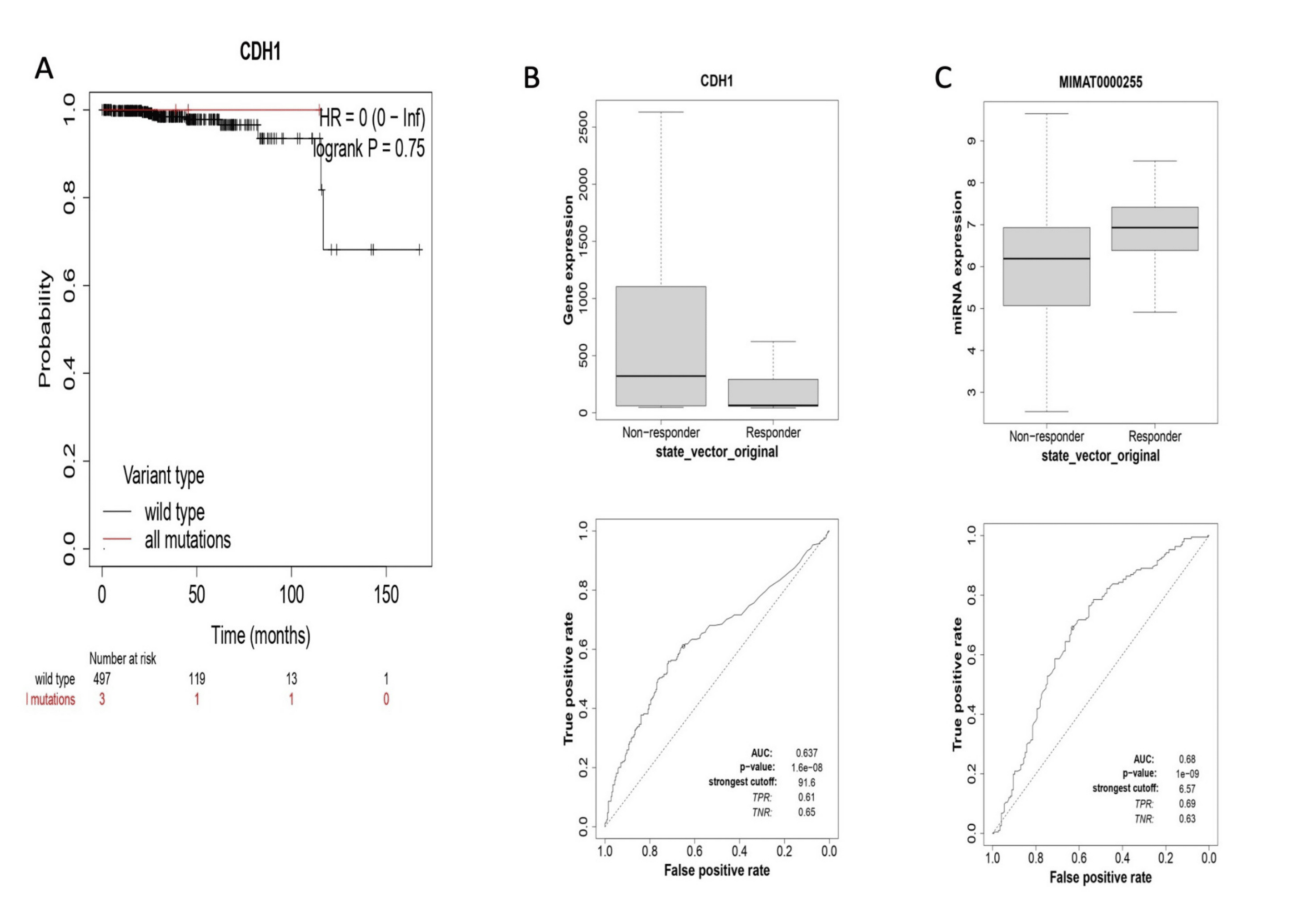
(A) Kaplan-Meier overall survival analysis of prostate cancer patients based on CDH1 gene expression. (B) Receiver operating characteristic (ROC) curve showing the diagnostic value of CDH1 in prostate cancer. (C) ROC curve showing the diagnostic value of miR-34a-5p in prostate cancer.

KM analysis revealed that high CDH1 mRNA expression correlated with improved overall survival in prostate adenocarcinoma patients (hazard ratio (HR): 0.67 (95% CI: 0.58-0.79), P < 0.0001), while comparison of wild-type versus mutated groups showed no survival difference (HR: 0, P = 0.75), with similar risk reduction trends in both groups (Figure 10A). CDH1 demonstrated strong diagnostic performance (area under the curve (AUC): 0.81-0.91, sensitivity: 0.81-0.92, specificity: 0.83-0.93) across gene expression thresholds (500-9000 units) (Figure 10B). MiR-34a-5p was identified as a significant biomarker (AUC 0.68, sensitivity 69%, specificity 63%), with MIMAT0000255 showing superior classification (AUC 0.88, sensitivity 89%, specificity 83%) when normalized using state_vector_original (Figure 10C).

## Discussion

EMT is critical for the progression and dissemination of prostate cancer. EMT is tightly regulated by multiple factors, including several miRs [12]. Prostate cancer malignancies from early to advanced stages have been linked to dysregulation of miR. We thoroughly analysed the expression of miR using the dbDEMC3.0 database (Zhejiang University, Hangzhou, China), evaluating 373 miR in prostate adenocarcinomas (of which 85 were significantly downregulated and 87 were significantly elevated).

Our in silico analysis of the differential expression of miR in vitro using DIANA-miTED revealed that, in comparison to SH-SY5Y (normal cell line), there was no significant change in miR-509-3-5p in the PC-3 and DU 145 cell lines. However, miR-141-3p, miR-200a-3p, miR-200b-3p, miR-200c-3p, and miR-429 were found to be significantly upregulated, while miR-34a-5p was found to be downregulated. It was observed that the expression of miR-34a was substantially lower in neighbouring benign tissues than in malignancies [13]. Some contradictory results were found regarding the increased miR-34a expression level achieved in prostate cancer samples compared to the initial counterpart [14]. The expression of miR-34a, an adequate tumor-suppressive miR, is dysregulated in prostate cancer. MiR-34a is a promising anti-prostate cancer stem cell therapy because it targets many molecules necessary for cancer stem cell survival and function, hence acting as a potent suppressor of cancer stem cells [15].

Using DIANA-plasmiR techniques, we found that miR-34a-5p was a good predictive and diagnostic biomarker in the current study. It was followed by miR-141-3p, miR-200a-3p, miR-200b-3p, miR-200c-3p, and miR-429. Our study identifies miR-34a-5p as a candidate diagnostic biomarker, consistent with its utility in pancreatic and breast cancers [16,17]. However, clinical translation necessitates addressing key limitations; for example, tumor heterogeneity (e.g., Gleason score stratification) may confound miR-target correlations; single-cell sequencing could resolve this, and TCGA’s bulk RNA-seq data may mask stromal-epithelial crosstalk, warranting spatial transcriptomics in future work. Mitchell et al. showed that miR-141 is a potential diagnostic marker of prostate cancer [18,11]. Moreover, miR-200a-3p, miR-200b-3p, miR-200c-3p, and miR-429 have been shown to be prognostic signatures validated in phase 2 cohorts of castration-resistant prostate cancer [19].

Furthermore, the expression levels of miR-141-3p, miR-200a-3p, miR-200b-3p, miR-200c-3p, miR-203a-3p, miR-429, miR-34a-5p, and miR-509-3-5p were validated using ENCORI in 495 prostate cancer patients and 52 normal samples. Among these, miR-141-3p, miR-200a-3p, miR-200b-3p, miR-200c-3p, miR-203a-3p, miR-429, and miR-34a-5p were upregulated in the prostate cancer patient sample compared to the control sample, whereas miR-509-3-5p did not show any significant change. Previous studies revealed that blood samples from two separate cohorts had higher levels of miR-141, miR-200a, and miR-200c expression than the control group [20]. Prostate cancer cell lines exhibit a considerable upregulation of miR-429 expression in comparison to normal prostate epithelial tissues [21]. It has been found that, in comparison to normal adjacent tissues, colorectal cancer tissues express miR-203a-3p at higher levels [22]. According to bioinformatic research, the expression level of miR-203a-3p in pancreatic tissues and cells was significantly lower than in normal pancreatic tissues and cells, despite some contradicting results [23]. When compared to adjacent control tissues and normal cell lines, the expression levels of miR-509-5p are downregulated in pancreatic tissue and pancreatic cell lines [24].

The MIENTURNET database found potential key miR target genes of the EMT among the DEmiR. The functional enrichment analysis for target genes and the networks of miR-target interactions showed that distinct miRs implicated in transcriptional dysregulation in cancer are critical among various cancer types, including prostate cancer. The miR-429, miR-34a-5p, miR-509-3-5p, miR-200a-3p, miR-200b-3p, miR-200c-3p, miR-203a-3p, and VIM, ZEB1, ZEB2, and CDH1 were identified to interact strongly with these miR. miR-203a-3p is one of the important miRs that target the genes CDH1, VIM, SNAI1, ZEB1, ZEB2, and EMT, according to the outcome of the network degree plot of the target gene and miR. Most of the miR identified ZEB2. Furthermore, the KM plotter and GEPIA results demonstrated that when prostate cancer progresses, there are changes in the expression of CDH1, CDH2, VIM, SNAI1, ZEB1, and ZEB2. Previous studies have revealed that prostate cancer tissue samples have substantially lower expression of CDH1 than benign prostatic hyperplasia (BPH) patient tissue samples [25]. Contradictory results were observed for GEPIA, which revealed that in prostate cancer, CDH1 was considerably increased in comparison to the control group. While our multi-database integration (GEPIA, ENCORI, DIANA tools) strengthens the validity of miR-34a-5p’s role in EMT regulation, we acknowledge contradictory reports on CDH1’s function in prostate cancer. For instance, CDH1’s upregulation in our analysis contrasts with its canonical tumor-suppressive role, a paradox observed in other cancers [9]. This discrepancy may reflect context-dependent roles during EMT progression, where compensatory feedback mechanisms transiently elevate CDH1 expression. Notably, miR-34a-5p’s downregulation could disrupt this balance, a hypothesis requiring experimental validation via luciferase assays or CRISPR-based models. It is well known that CDH1 suppresses tumor growth. The maintenance of epithelial integrity during prostate oncogenic transition, tumor start, and progression demonstrates the comprehensive significance of CDH1 (E-cadherin). It is yet unknown what role E-cadherin plays biologically in maintaining the integrity of the prostatic epithelium and the underlying molecular processes [26]. SNAI1 and CDH2 did not exhibit any appreciable changes in expression. ZEB2 and SNAI1 were shown to be substantially downregulated in the patient sample in comparison to the control sample. Past studies have shown that patients with prostate cancer who had lymph node metastases or high-grade primary tumors generally expressed more CDH2 than patients with low-grade tumors [27]. According to the contradictory findings, SNAI1 gene expression was higher in gastrointestinal tumor tissue than in normal tissues, and ZEB2 expression was significantly higher in prostate cancer tissue than in BPH tissue [28]. ZEB1 gene expression was previously found to be greater in gastric cancer tissues compared to nearby non-tumor or normal gastric tissues [29].

Further interaction and co-expression analyses were performed using ENCORI stage-specific progression, which revealed that miR-34a-5p is strongly correlated with EMT genes (CDH1, CDH2, SNAI1, ZEB1, and ZEB2). These findings showed a significant p-value of negative correlation, emphasizing that CDH1 is a putative target of miR-34a-5p in prostate cancer. A survival analysis of CDH1 revealed a strong correlation between the patient's gene expression and survival. Similarly, this type of study previously demonstrated the miR-200c-3p/ZEB2 regulatory loop in prostate cancer progression [30]. While our integrated bioinformatics approach identified miR-34a-5p/CDH1 as a putative regulatory axis, we acknowledge the paradoxical upregulation of CDH1 (a canonical tumor suppressor) in prostate adenocarcinoma. This may reflect context-dependent roles, such as epithelial maintenance in early-stage tumors [26] or post-translational inactivation [25]. Although miR-34a-5p shows moderate diagnostic potential (AUC: 0.68), independent clinical validation is required to confirm its utility. Future studies should combine mechanistic assays (e.g., luciferase reporters) with multi-cohort profiling to resolve these discrepancies.

A key limitation of this study is the need for further experimental validation and clinical confirmation of our findings. The lack of liquid biopsy data limits insights into non-invasive biomarker applicability. Future studies should integrate functional assays (e.g., luciferase reporters, patient-derived xenograft (PDX) models) and clinically annotated cohorts to assess translational potential. This study’s reliance on in silico predictions necessitates experimental validation. For instance, the miR-34a-5p/CDH1 interaction, while strongly correlated (p < 0.001), requires confirmation via knockdown and overexpression models. Additionally, the clinical relevance of CDH1 upregulation warrants investigation across tumor stages and subtypes.

## Conclusions

Our computational analysis identifies the miR-34a-5p/CDH1 axis as a novel regulatory mechanism in prostate adenocarcinoma, highlighting its potential as a biomarker and therapeutic target for EMT inhibition. Preliminary findings suggest miR-34a-5p may modulate CDH1-mediated epithelial integrity. Ongoing experiments using miR-34a-5p overexpression in CDH1-knockout cells are currently underway to confirm causality. Further clinical validation and standardized assays are needed for translation. This study reveals the critical role of dysregulated miRNAs, particularly miR-34a-5p, in prostate cancer progression via EMT. We uncover a regulatory network where miRNAs interact with EMT markers, influencing tumor aggressiveness and survival. To our knowledge, this is the first investigation of the miR-34a-5p/CDH1 loop in prostate cancer, offering new insights for biomarker discovery and targeted therapies, with CDH1 as a potential therapeutic target. While miR-34a-5p emerges as a candidate biomarker, its clinical adoption requires standardized detection in liquid biopsies and prospective trials. These findings should guide hypothesis-driven research rather than immediate therapeutic application.

## References

[1] Bray F, Laversanne M, Sung H, Ferlay J, Siegel RL, Soerjomataram I, Jemal A (2024). Global cancer statistics 2022: GLOBOCAN estimates of incidence and mortality worldwide for 36 cancers in 185 countries. CA Cancer J Clin.

[2] Gosselaar C, Roobol MJ, Schröder FH (2005). Prevalence and characteristics of screen-detected prostate carcinomas at low prostate-specific antigen levels: aggressive or insignificant?. BJU Int.

[3] Bartel DP (2018). Metazoan microRNAs. Cell.

[4] Volinia S, Calin GA, Liu CG (2006). A microRNA expression signature of human solid tumors defines cancer gene targets. Proc Natl Acad Sci U S A.

[5] Kumari S, Manoj A, Rungta S (2024). Discovery and validation of novel microRNA panel for non-invasive prediction of prostate cancer. Cureus.

[6] Schwarzenbach H, Nishida N, Calin GA, Pantel K (2014). Clinical relevance of circulating cell-free microRNAs in cancer. Nat Rev Clin Oncol.

[7] Xu L, Qi X, Duan S (2014). MicroRNAs: potential biomarkers for disease diagnosis. Biomed Mater Eng.

[8] Yamamura S, Saini S, Majid S, Hirata H, Ueno K, Deng G, Dahiya R (2012). MicroRNA-34a modulates c-Myc transcriptional complexes to suppress malignancy in human prostate cancer cells. PLoS One.

[9] Bruner HC, Derksen PW (2018). Loss of E-cadherin-dependent cell-cell adhesion and the development and progression of cancer. Cold Spring Harb Perspect Biol.

[10] Khan MI, Hamid A, Adhami VM, Lall RK, Mukhtar H (2015). Role of epithelial mesenchymal transition in prostate tumorigenesis. Curr Pharm Des.

[11] Taylor BS, Schultz N, Hieronymus H (2010). Integrative genomic profiling of human prostate cancer. Cancer Cell.

[12] Hussen BM, Shoorei H, Mohaqiq M, Dinger ME, Hidayat HJ, Taheri M, Ghafouri-Fard S (2021). The impact of non-coding RNAs in the epithelial to mesenchymal transition. Front Mol Biosci.

[13] Fang LL, Sun BF, Huang LR (2017). Potent inhibition of miR-34b on migration and invasion in metastatic prostate cancer cells by regulating the TGF-β pathway. Int J Mol Sci.

[14] Liang J, Li Y, Daniels G (2015). LEF1 targeting EMT in prostate cancer invasion is regulated by miR-34a. Mol Cancer Res.

[15] Li WJ, Liu X, Dougherty EM, Tang DG (2022). microRNA-34a, prostate cancer stem cells, and therapeutic development. Cancers (Basel).

[16] Alemar B, Izetti P, Gregório C (2016). miRNA-21 and miRNA-34a are potential minimally invasive biomarkers for the diagnosis of pancreatic ductal adenocarcinoma. Pancreas.

[17] Orangi E, Motovali-Bashi M (2019). Evaluation of miRNA-9 and miRNA-34a as potential biomarkers for diagnosis of breast cancer in Iranian women. Gene.

[18] Mitchell PS, Parkin RK, Kroh EM (2008). Circulating microRNAs as stable blood-based markers for cancer detection. Proc Natl Acad Sci U S A.

[19] Lin HM, Mahon KL, Spielman C (2017). Phase 2 study of circulating microRNA biomarkers in castration-resistant prostate cancer. Br J Cancer.

[20] Cheng HH, Mitchell PS, Kroh EM (2013). Circulating microRNA profiling identifies a subset of metastatic prostate cancer patients with evidence of cancer-associated hypoxia. PLoS One.

[21] Ouyang Y, Gao P, Zhu B (2015). Downregulation of microRNA-429 inhibits cell proliferation by targeting p27Kip1 in human prostate cancer cells. Mol Med Rep.

[22] Chen L, Gao H, Liang J (2018). miR-203a-3p promotes colorectal cancer proliferation and migration by targeting PDE4D. Am J Cancer Res.

[23] An N, Zheng B (2020). miR-203a-3p inhibits pancreatic cancer cell proliferation, EMT, and apoptosis by regulating SLUG. Technol Cancer Res Treat.

[24] Li X, Li Y, Wan L, Chen R, Chen F (2017). miR-509-5p inhibits cellular proliferation and migration via targeting MDM2 in pancreatic cancer cells. Onco Targets Ther.

[25] Jaggi M, Johansson SL, Baker JJ, Smith LM, Galich A, Balaji KC (2005). Aberrant expression of E-cadherin and beta-catenin in human prostate cancer. Urol Oncol.

[26] Olson A, Le V, Aldahl J (2019). The comprehensive role of E-cadherin in maintaining prostatic epithelial integrity during oncogenic transformation and tumor progression. PLoS Genet.

[27] Jaggi M, Nazemi T, Abrahams NA, Baker JJ, Galich A, Smith LM, Balaji KC (2006). N-cadherin switching occurs in high Gleason grade prostate cancer. Prostate.

[28] Fang J, Ding Z (2020). SNAI1 is a prognostic biomarker and correlated with immune infiltrates in gastrointestinal cancers. Aging (Albany NY).

[29] Fard SS, Sotoudeh M, Saliminejad K (2020). Investigation of the correlation between androgen receptor and ZEB1 and its value in progression of gastric cancer. Avicenna J Med Biotechnol.

[30] Zhang J, Zhang H, Qin Y, Chen C, Yang J, Song N, Gu M (2019). MicroRNA-200c-3p/ZEB2 loop plays a crucial role in the tumor progression of prostate carcinoma. Ann Transl Med.

